# Mathematical model of a serine integrase-controlled toggle switch with a single input

**DOI:** 10.1098/rsif.2018.0160

**Published:** 2018-06-06

**Authors:** Alexandra Pokhilko, Oliver Ebenhöh, W. Marshall Stark, Sean D. Colloms

**Affiliations:** 1 Institute of Molecular, Cell and Systems Biology, University of Glasgow, Glasgow G12 8QQ, UK; 2 Cluster of Excellence on Plant Sciences (CEPLAS), Heinrich-Heine-University, Universitätsstraße 1, 40225 Düsseldorf, Germany

**Keywords:** toggle switch, serine integrase, synthetic biology, binary counter, mathematical model

## Abstract

Dual-state genetic switches that can change their state in response to input signals can be used in synthetic biology to encode memory and control gene expression. A transcriptional toggle switch (TTS), with two mutually repressing transcription regulators, was previously used for switching between two expression states. In other studies, serine integrases have been used to control DNA inversion switches that can alternate between two different states. Both of these switches use two different inputs to switch ON or OFF. Here, we use mathematical modelling to design a robust one-input binary switch, which combines a TTS with a DNA inversion switch. This combined circuit switches between the two states every time it receives a pulse of a single-input signal. The robustness of the switch is based on the bistability of its TTS, while integrase recombination allows single-input control. Unidirectional integrase-RDF-mediated recombination is provided by a recently developed integrase-RDF fusion protein. We show that the switch is stable against parameter variations and molecular noise, making it a promising candidate for further use as a basic element of binary counting devices.

## 1. Introduction

Genetic switches with two states (ON/OFF) are essential components of synthetic biology memory and counting devices, with potential application in biotechnology, biosensors and biocomputing [1–3]. The creation of these binary switches is, therefore, an important goal of synthetic biology. Here, we design a synthetic genetic switch, which switches between two states in response to a *single-input signal*. The response of the switch depends on its current state. If it is OFF when it receives an input signal, it switches to ON; if it is ON, it switches to OFF. An orthogonal set of single-input state-based toggle switches with this behaviour could be used to encode the digits in a binary ripple counter [2]. In such a counter, each switch represents a single binary digit, and *N* interconnected switches would be able to count up to 2*^N^*−1 occurrences of the same repeated signal. The counting of various intracellular or extracellular events can then be used to control intracellular processes, to track genetic lineage, or to count the occurrences of events [2,4]. No single-input switch capable of robust toggling between two states has been implemented to date.

**Figure 1.**
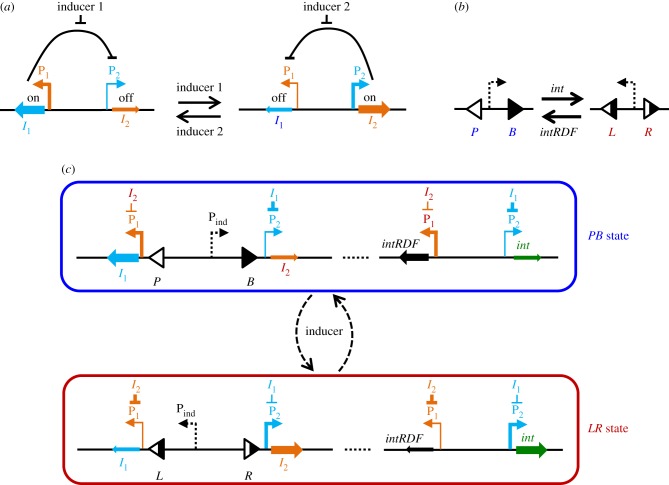
Gene circuit of integrase-controllable inversion-and-transcriptional toggle switch (ITTS). (*a*,*b*). Basic elements of the switch. (*a*) Two states of the bistable transcriptional toggle switch (TTS), expressing *I*_1_ (left) or *I*_2_ (right). The TTS is regulated by mutual repression of expression of *I*_1_ and *I*_2_ inhibitors from *I*_2_- and *I*_1_- regulated promoters (P_1_ and P_2_). Two different input signals (inducer 1 and 2) initiate the transition between the two states, by de-repressing the respective promoters. (*b*) A DNA inversion switch that can switch between two DNA states (*PB* and *LR*), mediated by serine integrase *int* and its fusion protein with RDF (*intRDF*), which invert the DNA fragment located between *P* and *B*, or *L* and *R* attachment sites. (*c*) Scheme of the one-input ITTS, illustrating the two states of the switch, expressing *I*_1_ and *intRDF* in the *PB* state (blue box) and *I*_2_ and *int* in the *LR* state (red box). The switch between states is initiated by a pulse of an inducer, activating the inducible promoter P_ind_. This results in the expression of the currently unexpressed inhibitor, followed by the expression of *int* (or *intRDF*) and changing of the DNA state.

The best-characterized bistable switch is the toggle switch, based on mutual repression of two inhibitors [5–8]. **Transcriptional toggle switches** (hereafter called **TTS**) are constructed *in vivo* and, therefore, can be directly used for intracellular applications. A TTS is based on the expression of two transcriptional repressors *I*_1_ and *I*_2_ [5,6,8]. Each repressor is expressed from a promoter repressed by the other repressor (**P_1_** or **P_2_**) (figure 1*a*), so that when *I*_1_ is expressed, transcription of *I*_2_ is turned off and vice versa. There are two steady states, with either *I*_1_ or *I*_2_ expressed. The switch between these two steady states can be brought about using two different inducers (input signals), such as IPTG and anhydrotetracycline (aTc), inducing transcription of the unexpressed repressor (figure 1*a*) [5]. Experimentally implemented TTS shows robust switching with two inputs [5,6]. However, the only single-input switch implemented to date, which combines a TTS and a logic gate, showed a damped response to repeated induction of the circuit [8].

Another class of genetic switch uses site-specific recombinases, enzymes that cut and re-join DNA at specific recombination sites. Depending on the arrangement of these sites in the DNA, recombinases carry out fusion, deletion or inversion reactions. Inversion of a DNA segment flanked by two recombination sites in a ‘head-to-head' orientation allows repeated switching between two alternative states. Placing a promoter on the invertible segment allows switching between expressions of two different genes (figure 1*b*). This has been used to make simple **inversion switches** that control gene expression, encode memory or carry out logical calculations [3,4,9–11]. Using serine integrases (***int***) for these genetic switches has the advantage of unidirectional recombination, and the ability to reverse this directionality by the addition of a recombination directionality factor (**RDF**) [10,12,13]. *Int* on its own carries out recombination on two specific DNA sequences called *attP* and *attB* sites (***PB***), producing *attL* and *attR* product sites (***LR***), each consisting of half of a *P* and half of a *B* site (figure 1*b*). The presence of the RDF reverses *int* directionality, so that *LR* recombines back to *PB*.

Previous switches used two inputs to control separate expression of *int* and *int*+RDF [10]. In this paper, we aim to design a robust single-input switch, which can be further used as a basic element of counters and memory devices. Our switch is based on a combination of two double-input switches (a TTS and a DNA inversion switch). The TTS, based on two mutually repressing inhibitors, controls whether *int* or *int*+RDF is synthesized (figure 1*c*). Expression of *int* or *int*+RDF in turn operates a DNA inversion switch, changing the orientation of an inducible promoter. Activation of the promoter by inducer (***ind***) provides a single-input signal, inducing expression of the currently inactive inhibitor and thus changing the state of the switch. We use mathematical modelling to demonstrate that the inversion-and-transcriptional toggle switch (ITTS) is capable of robust switching between two DNA states over a broad range of parameters and is stable against molecular noise. We anticipate that the robustness of the switch should make it useful for further experimental implementations of single-input memory devices.

## 2. Model description

Here, we use mathematical modelling to develop a single-input DNA switch, the ITTS. Similar to previous work, our switch is designed to be implemented in *Escherichia coli* cells bearing plasmids with the switch gene circuit [10]. The ITTS integrates a TTS (figure 1*a*) and a DNA inversion switch operated by *int* and its RDF (figure 1*b*). It has been shown recently that *LR*-to-*PB* recombination is more efficient with an integrase-RDF fusion protein (*intRDF*). This fusion protein improves directionality compared to a mixture of separate *int* and RDF proteins, and expression of a single protein simplifies the switch design [14] (figure 1*b*). Our ITTS, therefore, uses *intRDF* to switch from *LR* to *PB*, and *int* to switch from *PB* to *LR*.

The TTS consists of two mutually repressing transcriptional inhibitors *I*_1_ and *I*_2_ expressed from P_1_ and P_2_ promoters (figure 1*a*). The *int* and *intRDF* genes are expressed from their own copies of the P_2_ and P_1_ promoters respectively, thus coupling the state of the inversion switch to the state of the TTS (figure 1*c*). When *I*_1_ is expressed and *I*_2_ is not, only *intRDF* will be expressed, putting the switch in the *PB* state (figure 1*c*, top). Similarly, when *I*_2_ is expressed, only *int* will be expressed and the switch will be in the *LR* state (figure 1*c*, bottom). Our switch design is not specific to any particular types of repressors *I*_1_ and *I*_2_. However, an essential requirement is that in order for the toggle switch to be bistable, the repressors have to bind their target promoters with cooperativity [5].

Switching between the two states of the ITTS is provided by periodic pulses of inducer *ind*, activating an inducible promoter P_ind_ located between *att* sites of the DNA inversion switch (figure 1*c*). For example, the sugar arabinose could be used as *ind* to induce the arabinose-inducible P_BAD_ promoter [15]. Experimentally, we envision testing the system using short 1–4 h pulses of inducer every 24 h. Therefore, we model *ind* mathematically using a suitable periodic function.

The orientation of P_ind_ depends on the state of the inversion switch, which in turn is governed by the TTS (figure 1*c*). When *I*_1_ is on, induction of P_ind_ will turn on expression of *I*_2_; when *I*_2_ is on P_ind_ will express *I*_1_. Each pulse of inducer results in a cycle of events: (i) P_ind_-mediated transient expression of the currently repressed inhibitor; (ii) a change in the state of the TTS (switch from *I*_1_ to *I*_2_ or *I*_2_ to *I*_1_ expression); and (iii) a switch between *int* and *intRDF* expression, and thus a change in the orientation of the invertible DNA segment.

### 2.1. Model equations

The intracellular kinetics of *int*, *intRDF*, *I*_1_ and *I*_2_ protein production and decay is described by four ordinary differential equations (ODEs), corresponding to the scheme of figure 1*c*. Based on fast mRNA degradation [16,17], we assumed that mRNA levels are proportional to promoter activities. Therefore, the rates of protein expression are simply proportional to promoter activities. All proteins were assumed to be diluted due to cell growth and division. The equations for *int*, *intRDF*, *I*_1_ and *I*_2_ proteins are as follows:2.1
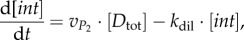
2.2
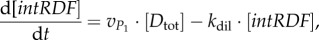
2.3
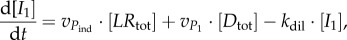
2.4
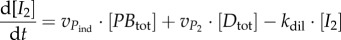

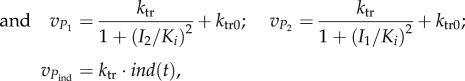
where [*int*] and [*intRDF*] are the concentrations of *int* and *intRDF* fusion protein; [*I*_1_]*,* [*I*_2_] are the concentrations of *I*_1_ and *I*_2_; and [*PB*_tot_] and [*LR*_tot_] are the concentrations of plasmid DNA in the *PB* and *LR* state, respectively, determined by the recombination reactions described below. [*D*_tot_] is the total concentration of plasmid DNA ([*D*_tot_] = [*PB*_tot_] + [*LR*_tot_]). 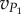, 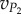 and 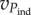 are the rates of protein expression from P_1_, P_2_ and P_ind_, respectively. Orthogonal inhibitors from the TetR family [18] represent likely candidates for *I*_1_ and *I*_2_ in future experimental implementation of the ITTS. Therefore, based on the reported dimeric structure of TetR complexes [19], we used a Hill coefficient of 2 for the inhibition of P_1_ and P_2_ by *I*_2_ and *I*_1_.

The recombination reactions implementing the conversion between the *PB* and *LR* states are described based on our minimal model of *in vitro* recombination by ϕC31 integrase with or without RDF (electronic supplementary material, figure S1) [20]. To describe *in vivo* recombination, we have included in the present model the dilution of *int* and *intRDF* proteins from their complexes with DNA upon DNA replication (equations (2.5) and (2.6)). Additionally, because we use *intRDF* fusion protein instead of a mixture of *int* with RDF, our model does not have the equation for the formation of the complex between *int* and RDF, which was used in [20].

The equations for recombination reactions were derived in [20] assuming that recombination steps (*r*1, *r*2) and synaptic conformational change steps (*syn, synr*) are much slower compared to other steps. The slow-changing variables *LRint*_1_, *PBintRDF*_1_ and *PB*_tot_ (sum of all *PB*-containing complexes) are described by three ODEs (electronic supplementary material, figure S1):2.5
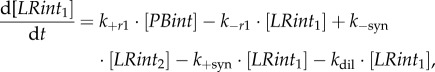
2.6
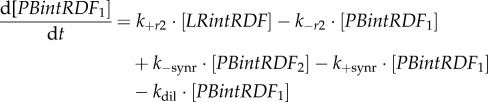
2.7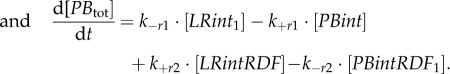 The algebraic equations for fast-changing variables were derived using rapid equilibrium approximations [20]:2.8
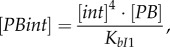
2.9
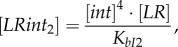
2.10
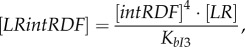
2.11
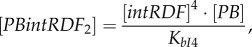
2.12
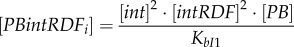
2.13
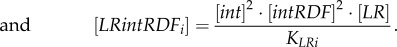


Free *PB* and *LR* concentrations were expressed from the mass balance equation for the *PB*- and *LR*-containing species [20]:2.14
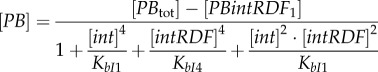
2.15
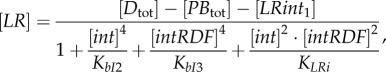
where [*PBint*]*,* [*LRintRDF*]*,* [*LRint*_1_]*,* [*LRint*_2_]*,* [*PBintRDF*_1_]*,* [*PBintRDF*_2_], [*PBintRDF_i_*]*,* [*LRintRDF_i_*] are the concentrations of the respective complexes and [*PB*], [*LR*] are the concentrations of free *PB* and *LR* DNA (electronic supplementary material, figure S1). [*PB*_tot_] and [*LR*_tot_] are the sums of all LR- and PB-containing complexes ([*LR*_tot_] *+* [*PB*_tot_] *=* [*D*_tot_]), respectively. *K_bI_*_1_, *K_bI_*_2_, *K_bI_*_3_, *K_bI_*_4_, *K_LRi_* are the dissociation constants for the respective complexes (*K_bI_*_1_, *K_bI_*_2_, *K_bI_*_3_, *K_bI_*_4_ are assumed to be equal to *K_bI_*). The parameters *k_+r_*, *k_+_*_syn_, *k_+_*_synr_, and *k_−r_*_1_, *k_−r_*_2_, *k_−_*_syn_, *k_−_*_synr_ stand for the forward and reverse rate constants of the slow recombination and synapsis (*syn*, *synr*) steps [20] (assuming *k_+r_*_1_ = *k_+r_*_2_ = *k_+r_*), with the forward direction defined as *PB* → *LR* for the *int* reaction and as *LR* → *PB* for the *intRDF* reaction (electronic supplementary material, figure S1) [20].

All concentrations are expressed in μM; the time units are hours.

### 2.2. Behaviour of the model components

*I*_1_ and *intRDF* proteins are expressed from copies of P_1_, while *I*_2_ and *int* are expressed from P_2_ promoters (figure 1*c*, equations (2.1)–(2.4)). The activities of P_1_ and P_2_ (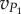 and 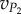) are sums of two terms: the main activity, which is inhibited by *I*_2_ and *I*_1_, respectively, and the promoter leakages (background activities in the presence of saturated concentrations of inhibitors). The expression of *I*_1_ and *I*_2_ is also transiently induced from P_ind_ during pulses of the external signal *ind*(*t*). Expression of *I*_1_ and *I*_2_ is described as a sum of the expression from P_ind_ and from P_1_ or P_2_ (equations (2.3) and (2.4)). This assumption is based on observations of additive gene expression from tandem promoters [21,22]. We assume that transcription initiated by P_ind_ can read through the repressor-bound P_1_ and P_2_ [21].

The recombination mechanisms are described in detail in [20]. Briefly, *PB*-to-*LR* recombination starts from binding of four molecules of *int* to the *PB* substrate (binding step *bI*_1_; electronic supplementary material, figure S1), followed by recombination (strand exchange, step *r*1) leading to formation of the product synaptic complex *LRint*_1_. The *LRint*_1_ complex can also slowly de-synapse to form *LRint*_2_ complex (step *syn*), which can dissociate and release free *LR* product (step *bI*_2_). The last two steps are unfavourable (electronic supplementary material, figure S1) and *LRint*_1_ represents the main form of the *LR* product *in vitro* [20]. However, *in vivo* dissociation of integrase from this stable product during DNA replication might increase the amount of free DNA. In our model, this is described through a dilution of *int* from *LRint*_1_ (equation (2.5)), which decreases *LRint*_1_ concentration and thus increases free *LR* product (equation (2.15)). This increases the recombination efficiency of *in vivo* reactions (§3.1). Similarly, *LR*-to-*PB* recombination starts from binding of four molecules of *intRDF* to the *LR* substrate (step *bI*_3_), followed by recombination (step *r*2) and the formation of the product synaptic complex *PBintRDF*_1_. The unfavourable steps include de-synapsis of *PBintRDF*_1_, producing *PBintRDF*_2_ (step *synr*) and release of the free *PB* product (step *bI*_4_). Dilution of *intRDF* from *PBintRDF*_1_ (equation (2.6)) decreases *PBintRDF*_1_ concentration and thus increases free *PB* product (equation (2.14)). The model also includes unproductive complexes *LRintRDF_i_* and *PBintRDF_i_* (equations (2.12), (2.13)), which form due to competition between *int* and *intRDF* dimers [20].

### 2.3. Simulation of the inversion-and-transcriptional toggle switch model

The system of ODEs was solved using MATLAB, integrated with the stiff solver ode15 s (MathWorks, Cambridge, UK). The MATLAB code of the model is provided in electronic supplementary material, text S1).

The total DNA concentration was taken to be 10 nM, based on a typical plasmid copy number (approx. 10 plasmids cell^−1^) and an estimated concentration of approximately 1 nM for one molecule/cell (based on a typical cell volume of approx. 1.6 × 10^−15^ l). The *K_i_* of promoter inhibition is set at 10 nM [23]. The effective rate constant of maximal protein production is estimated as *k*_tr_ = 360 h^−1^ [16,17]. The rate constant of background protein production due to leakages from repressed promoters (in the presence of a saturated concentration of the inhibitor) was assumed to be *k*_tr0_ = 3.6 h^−1^ [16,17]. As transcription and translation are described by a single step in our model, the effects of promoter and ribosome-binding site strengths are not distinguishable and were varied in the model by changing the rate constant of protein production. The rate constants of *I*_1_, *I*_2_, *int* and *intRDF* protein production were assumed to be equal to *k_tr_* in all simulations, except those where the rates of *int* or *intRDF* production were separately varied, as stated in the text. *k*_dil_ was determined from the characteristic doubling time of 20 min for fast-growing culture.

The input signal was simulated using a previously developed periodic step function *ind*(*t*) [24], mimicking periodic addition and withdrawal (e.g. by dilution of the cell culture) of the inducer2.16
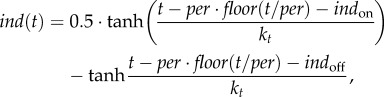
where *ind*_on_ and *ind*_off_ determine the times of the beginning and end of each pulse of inducer, administrated with a period *per* (*per* is chosen to be 24 h for the convenience of the future experimental design); *k_t_* is a characteristic time of the inducer's decay (*k_t_* = 0.3 h based on a 20 min cell doubling time).

The equilibrium constants of recombination reactions satisfy the energy conservation equations for *PB*-to-*LR* and *LR*-to-*PB* transitions (electronic supplementary material, figure S1) [20]:
2.17
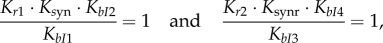
where *K_r_*_1_, *K_r_*_2_, *K*_syn_, *K*_synr_ are the equilibrium constants (*k_+_/k_−_*) of the respective steps and *K_bI_*_1_, *K_bI_*_2_, *K_bI_*_3_, *K_bI_*_4_ are the dissociation constants (*k_−_/k_+_*, where *k_+_* and *k_−_* are rate constants of binding and dissociation of integrase or *intRDF* from DNA). The modelling of *int* with reduced efficiency (§3.2) was done by decreasing the equilibrium constants of the recombination steps *K_r_*_1_, *K_r_*_2_ 10-fold, with compensating 10-fold increases of the dissociation constants *K_bI_*_2_, *K_bI_*_4_ of *int* binding to DNA products, to comply with energy conservation (equation (2.17)). The model parameters are presented in electronic supplementary material, table S1.

## 3. Results and discussion

During the construction of the ITTS, we initially considered a simpler scheme with *int* and *intRDF* expressed from a constitutive promoter in an invertible DNA segment (electronic supplementary material, figure S2). The switch was expected to be bistable due to the expression of *intRDF* in the *PB* state, converting any *LR* product back to *PB* and expression of *int* in the *LR* state, maintaining the DNA in the *LR* state. This switch would operate by induction of expression of *int* or *intRDF* from an oppositely oriented inducible promoter within the invertible DNA segment. However, we found that the switch could not alternate between the two states in response to inducer pulses. Instead, over a broad parameter range, the switch always ends up in the *LR* state, due to the higher efficiency of *PB*-to-*LR* conversion. The inability to switch state was caused by rapid initiation of recombination during the inducer pulse, leading to overlapping production of *int* and *intRDF* proteins. In order for the switch to make reliable transitions on inducer pulse, expression of *int* and *intRDF* from the inducible promoter must be temporally distinct from integrase-mediated inversion. This is difficult to achieve due to the rapid nature of transcriptional induction and site-specific recombination. The simultaneous expression of *int* and *intRDF* is avoided in our final design (figure 1*c*) due to the tight control of *int* and *intRDF* expression by the TTS, as described below.

### 3.1. The kinetics of the inversion-and-transcriptional toggle switch

**Figure 2.**
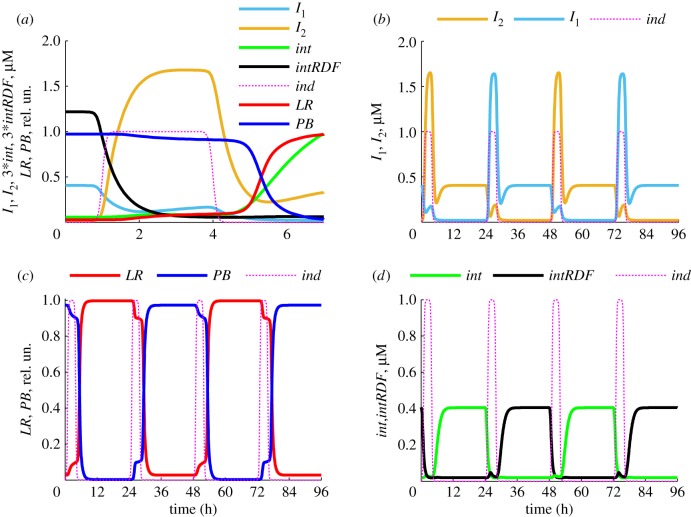
Intracellular kinetics of the ITTS. (*a*) The concentrations of *I*_1_ (light blue), *I*_2_ (orange), *int* (green), *intRDF* (black) and the relative (normalized to total) concentrations of *LR* (red) and *PB* (blue) DNA during the first hours of the *PB*-to-*LR* transition. (*b–d*) The long-term kinetics of the ITTS, with 3-h pulses of inducer repeated every 24 h. (*b*) The concentrations of *I*_1_ (blue) and *I*_2_ (orange). (*c*) The relative concentrations of *LR* (red) and *PB* (blue). (*d*) The concentrations of *int* (green) and *intRDF* (black). The inducer kinetics (in relative units) is shown on all panels by magenta dotted lines. The half-time of inducer decay is *k_t_* = 0.3 h on (*b*–*d*) and *k_t_* = 0.1 h on (*a*), for sharper transition (for clarity of the figure). All calculations were done for the equal strengths of P_ind_, P_1_ and P_2_.

The model of our single-input switch ITTS is described in §2 (figure 1*c*). The switch has two steady states (§3.2) and is capable of robust switching between the two states, as we show below. The single-input signal to the ITTS is provided by pulses of an external inducer, described by periodic step function *ind(t)* (equation (2.16)). Surprisingly, the model predicts that the switch of the DNA state is completed only after the inducer pulse finishes, due to the interactions between the ITTS components. Thus, if the switch was initially in the *PB* state, expressing *I*_1_ and *intRDF* (figure 1*c* top; figure 2*a*), then the addition of inducer causes an increase of *I*_2_, which downregulates *I*_1_ and *intRDF* expression from the *I*_2_-inhibited P_1_ promoters. Decreased expression results in decreased protein levels, due to protein dilution during cell growth and division. The initial decrease in *I*_1_ initiates a minor increase of *int* (figure 2*a*). The decrease of the *intRDF*/*int* ratio causes slight increase of *LR* (at approx. 2 h on figure 2*a*, when *int* ∼ *intRDF*), but in the presence of inducer this leads to a secondary wave of *I*_1_ expression from the P_ind_ promoter in the *LR* state. This prevents further increase of the *int* concentration and thus *PB*-to-*LR* conversion (figure 2*a*). Under induction with relatively strong P_ind_ (figure 2), concentrations of both inhibitors are high enough during the pulse to prevent production of *int* and *intRDF*. Therefore, the *PB*-to-*LR* transition is completed only after the inducer pulse finishes (figure 2*a*). *I*_1_ and *I*_2_ both decrease after the pulse, but the TTS falls into the *I*_2_ steady state because 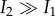 (figure 2*a*). The concentration of *int* is initially low after the pulse; it starts to increase only when *I*_1_ falls below the critical level required for the release of the repressed P_2_ promoter (half-released at 0.01 µM [23]). The inversion switch follows the TTS after the minimal *int* concentration required for recombination (0.1 µM [25]) is achieved (approx. 5 h on figure 2*a*). When the ITTS is in the *LR* state, a pulse of inducer produces a switch to *PB* by a similar mechanism due to the symmetry of the ITTS design (figure 1*c*, figure 2*b–d*).

*Int* recombination efficiencies observed experimentally *in vivo* [14] are typically higher than those observed *in vitro* [25]*.* Our previous models for *int* recombination [20,25] fit the *in vitro* data, predicting 80% and 70% recombination of *PB*-to-*LR* and *LR-*to-*PB*, respectively. To mimic the *in vivo* situation, the model was modified to include stripping of *int* and *intRDF* from DNA during DNA replication, accelerating the release of free DNA from reaction products (§2; electronic supplementary material, figure S1). The modified model predicts highly efficient intracellular conversion of *PB*-to-*LR* and *LR-*to-*PB* (100% and 97%, respectively) (figure 2*c*), in agreement with the *in vivo* data.

### 3.2. The robustness of the inversion-and-transcriptional toggle switch to parameter variations

Two characteristics are important for the ITTS operation: (i) coexistence of two steady states in the absence of inducer (bistability) and (ii) ability to switch between the two states in response to the inducer pulse. The bistability of the ITTS is determined by the TTS parameters, while the ability to switch depends on the parameters of P_ind_ induction (pulse duration and P_ind_ strength) and parameters of the inversion switch, as discussed below.

**Figure 3.**
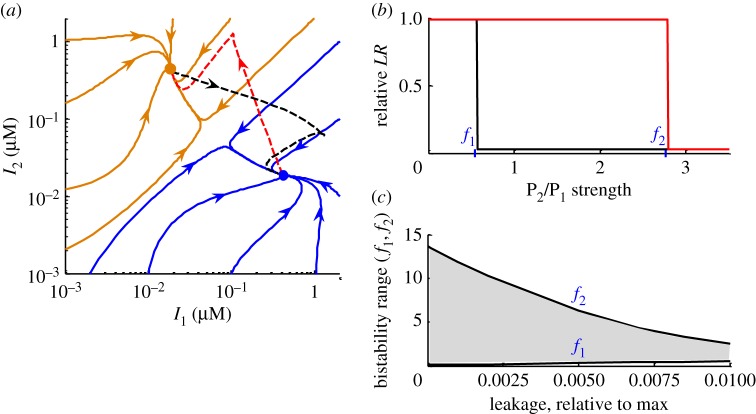
Bistability range of the ITTS. (*a*) Phase diagram of *I*_1_ and *I*_2_ trajectories, starting from different *I_1_* and *I*_2_ concentrations, with arrows showing the direction of the time. All trajectories end up in one of two steady states with high *I*_1_ or high *I_2_* (indicated by blue and orange dots, respectively). Black and red trajectories show the transitions between steady states after the addition of inducer (1 h pulse). (*b*) Dependence of the steady-state *LR* levels (normalized to total DNA) on the fold difference in the strength of P_2_ relative to P_1_. Two steady states with high and low LR levels are shown by red and black lines, respectively. The lower and upper margins of the bistable region are marked by the symbols f_1_ and f_2_, respectively. (*c*) Dependence of the bistability range (values of f_1_ and f_2_) on the values of P_1_ and P_2_ leakages. Graphs in (*a*,*b*) were calculated with leakages in P_1_ and P_2_ equal to 1% of maximal activity. All calculations, except black and red dashed lines in (*a*), were done in the absence of inducer.

The bistability of the ITTS is based on the bistability of its TTS. Figure 3*a* shows the ITTS dynamics in the absence of inducer on a phase diagram, showing trajectories in the *I*_1_/*I*_2_ phase plane. Different initial concentrations of *I*_1_ and *I*_2_ produce different trajectories, and all the trajectories end up in one of the two stable steady states with high *I*_1_ (blue) or high *I*_2_ (orange) concentrations. We used the model to explore the dependence of the bistability range on the strengths of P_1_ and P_2_ promoters. The simulations were run in the absence of inducer, starting from different initial concentrations of *I*_1_ and *I*_2_ (as on figure 3*a*). Both maximal activities and leakages (background expression from fully repressed promoter) affect the bistability range. When leakages in P_1_ and P_2_ promoters are relatively high (1% of the activities of unrepressed promoters), bistability is observed only for relatively similar promoter strengths (up to 2.5-fold difference in P_1_ and P_2_ strengths; figure 3*b*). The promoters of the TetR family have relatively high leakages and similar strengths [18], and so could be appropriate. Additionally, the ITTS is predicted to maintain its bistability when the promoters have substantially different strengths, providing that leakages are low. Thus, a 10-fold decrease in P_1_ and P_2_ leakages extends the bistability range up to 10-fold difference in P_1_ and P_2_ strengths (figure 3*c*). We conclude that the ITTS is bistable over a broad parameter range of promoter strengths and leakages.

**Figure 4.**
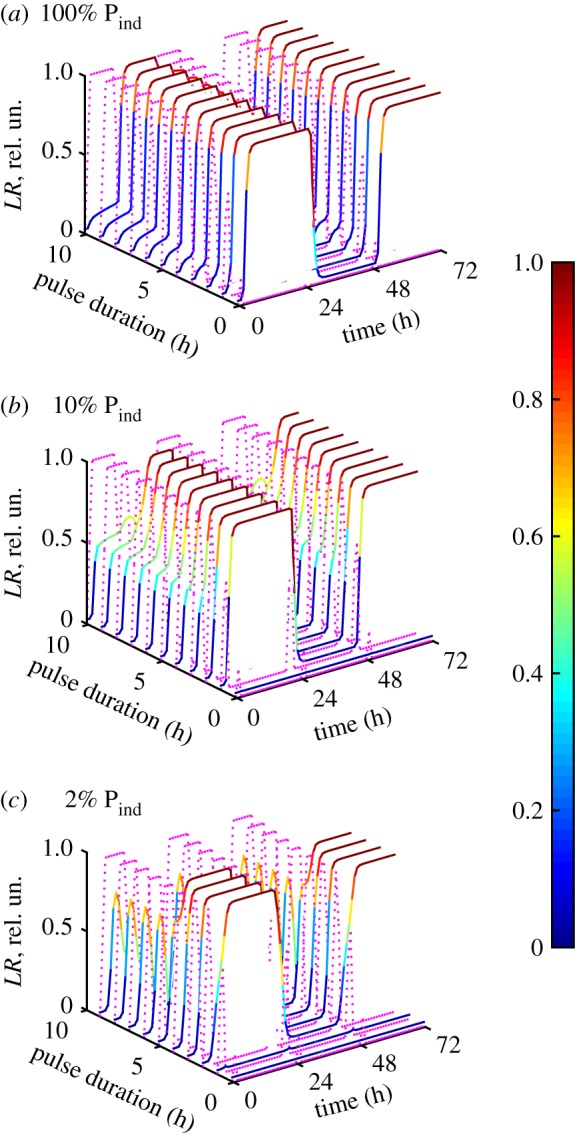
Dependence of the ITTS kinetics on the duration of inducer pulse and P_ind_ strength. The inducer kinetics is shown in magenta dotted lines, and *LR* kinetics is shown with a colour gradient (values are on colour bars), for different pulse durations. Computations were done for 100% (*a*), 10% (*b*) and 2% (*c*) strength of P_ind_ relative to P_1_ and equal strengths of P_1_ and P_2_. The strength of P_ind_ (relative to P_1_) is shown on each panel. The duration of the first, shortest pulse is 6 min, with subsequent plots for pulse lengths increasing at 1 h intervals.

In addition to being bistable, the ITTS is able to switch between the two states in response to the addition of inducer, as shown in figure 3*a* by black and red dashed lines. Figure 4 shows that the ITTS is capable of operating over a broad range of inducer pulse lengths and strengths of P_ind_. Thus, for a relatively high strength of the P_ind_ promoter (P_ind_ strength greater than 20% of P_1_ strength, with equal strengths of P_1_ and P_2_), the ITTS operates in both directions with any duration of inducer pulse longer than 4 min (figure 4*a*) and the DNA transitions happen only after the inducer pulse finishes, as described in §3.1. Therefore, a switch with strong P_ind_ promoter is not sensitive to pulse duration. However, reduction of the P_ind_ strength narrows the range of useful inducer pulses. Thus, for a P_ind_ with 10% of the strength of P_1_ and P_2_, the inducer pulse duration required for the efficient switching is between 0.5 and 9 h (figure 4*b*). For a P_ind_ with 2% of the P_1_ strength, the range of effective pulses narrows to 3–5 h (figure 4*c*).

**Figure 5.**
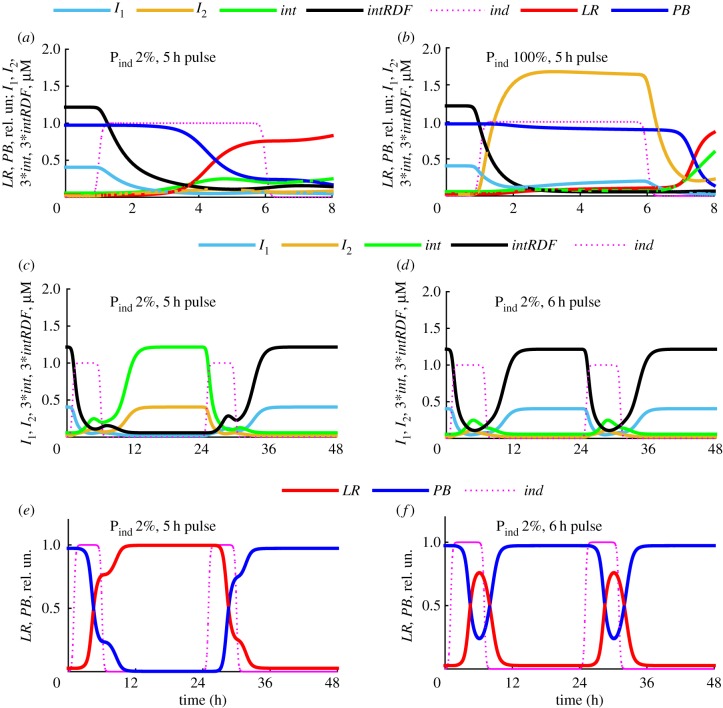
Dependence of ITTS kinetics on P_ind_ strength. (*a*,*b*) The concentrations of *I_1_* (light blue), *I_2_* (orange), *int* (green), *intRDF* (black) and the relative concentrations of *LR* (red) and *PB* (blue) during the first hours of the *PB*-to-*LR* transition, for P_ind_ promoter strengths of 2% (*a*) and 100% (*b*) relative to P_1_. (*c*–*f*) The long-term kinetics of the ITTS for 2% P_ind_ strength and pulse lengths inside (*c*,*e*) and outside (*d*,*f*) the functional range. Inducer pulse durations are 5 (*a*–*c*,*e*) and 6 (*d*,*f*) hours. (*c,d*) The concentrations of *I*_1_ (blue), *I*_2_ (orange), *int* (green) and *intRDF* (black). (*e*,*f*) The relative concentrations of *LR* (red) and *PB* (blue) DNA. The strengths of P_1_ and P_2_ are equal. The inducer kinetics are shown on all panels by magenta dotted lines.

The narrower range of permitted pulse lengths with a weak P_ind_ is due to low and comparable concentrations of the induced inhibitors during the pulse (figure 5*a,b*). Thus, if the ITTS was initially in the *PB* state, *I*_2_ is induced by *ind* (figure 5*a*), but to much lower levels than with the strong P_ind_ (figure 5*b*). *I*_1_ slowly decreases, increasing the *int* to *intRDF* ratio and initiating the *PB*-to-*LR* transition (figure 5*a*). *I*_1_ is expressed from P_ind_ in the *LR* state, but only to low levels compared to the strong P_ind_ (figure 5*a*,*b*), allowing near-complete transition to the *LR* state during a long pulse (figure 5*a*,*e*). The conversion to LR causes *I*_1_ concentration to increase again (figure 5*c*,*d*). For long enough pulses, *I*_1_ eventually becomes higher than *I*_2_ (figure 5*d*), reverting the transition back to the *PB* state (figure 5*f*). For shorter pulses, *I*_1_ remains lower than *I*_2_ throughout the pulse (figure 5*a*), allowing the TTS to complete the transition to *LR* after the pulse (figure 5*e*).

**Figure 6.**
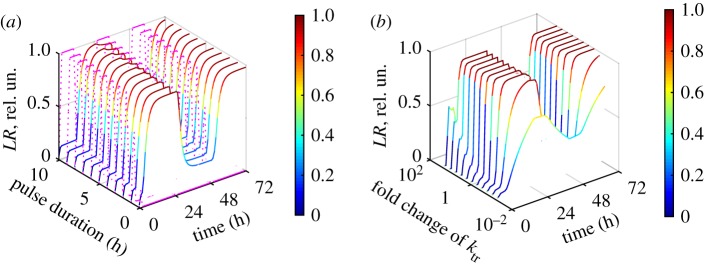
Effects of the parameters of DNA inversion on the ITTS kinetics. The *LR* kinetics is shown with a colour gradient. (*a*) The kinetics of the ITTS under different durations of inducer pulse, for low-efficient *int* and *intRDF*, simulated by 10-fold decrease of the equilibrium constants of recombination steps *K_r_*_1_, *K_r_*_1_. Computations were done for equal strengths of P_ind_, P_2_ and P_1_. The inducer kinetics is shown by magenta dotted lines. (*b*) Dependence of the ITTS kinetics on the fold change in the rate constant of *int* and *intRDF* production (*k_tr_* in terms *v_P_*_1_, *v_P_*_2_ of only equations (2.1), (2.2)), for an inducer pulse of 4 h duration.

Next, we explored the effect of the parameters of DNA inversion on the ITTS operation. Figure 6*a* shows the operation of the ITTS with low-efficiency *int* and *intRDF*, simulated by 10-fold decreases in the equilibrium constants of the recombination steps (*K_r_*_1_ and *K_r_*_2_). The efficiency of conversion from *LR* to *PB* with these altered parameters is reduced to 79% (compared with 97% with the high-efficiency *int* and *intRDF*), while the *PB*-to-*LR* conversion is reduced from 100 to 97% (figure 6*a*). However, switching between the two states is still robust over a broad range of pulse durations (figure 6*a*).

In addition to the variations in the efficiency of *int*-mediated recombination, the inversion switch might be affected by the expression rates of *int* and *intRDF*. However, our analysis demonstrates that the ITTS operates over a broad range (approx. 100-fold variation) of *int* and *intRDF* production rates (figure 6*b*). Very low rates of *int* and *intRDF* expression were insufficient to promote transition between the *PB* and *LR* states. Excessive levels of *int* and *intRDF* expression led to more than 50% transition during the pulse (electronic supplementary material, figure S3). This reduced the working range of pulse durations by the same mechanism as for low P_ind_ (figure 5), due to competition between the two inhibitors expressed from P_ind_ in the *PB* and *LR* states.

We conclude that the ITTS is very stable against variation in the parameters of the recombination reactions, in contrast to a previously developed inversion switch [10]. This is due to the coupling of the inversion switch to the bistable TTS in our ITTS design, ensuring that only one of *int* and *intRDF* proteins is expressed (figure 2*d*). In addition, the inversion switch is stabilized by the use of the *intRDF* fusion protein, increasing the efficiency of the *LR*-to-*PB* transition compared to a mixture of integrase and RDF [10].

The ITTS is designed to be implemented in *Escherichia coli* cells. In each cell, the circuit is predicted to switch efficiently between the two states in response to each inducer pulse. However, due to potential differences in initial conditions when the circuit is first introduced into cells, the switch might start in the *PB* state in some cells and the *LR* state in others. Therefore, in future experimental implementations of the ITTS, the cells might need to be synchronized initially by adding an inducer to activate either P_1_ or P_2_ [26] (figure 1*a*).

The switch can be used to express different genes, depending on the desired applications. For example, expression of two different fluorescent reporters (e.g. GFP and RFP) in the two switch states would allow monitoring of the switch kinetics. Alternatively, the switch could be used to control expression of further integrases to build more complex circuits, for instance, a ripple counter as discussed in the Introduction and Conclusion.

### 3.3. Effects of molecular noise

**Figure 7.**
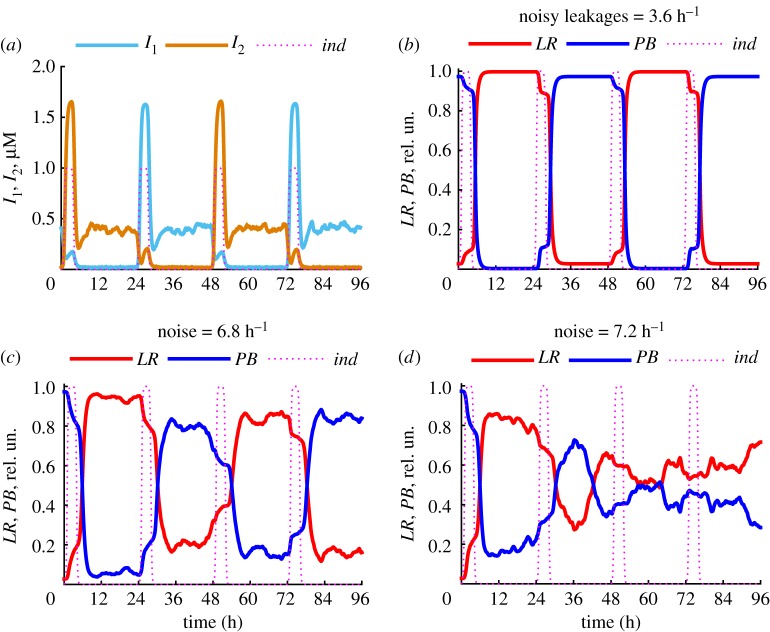
Stability of the ITTS to molecular noise. (*a*) Typical kinetics of *I*_1_ and *I*_2_ concentrations, calculated with the level of Poisson noise in P_1_ and P_2_ leakages, equivalent to the leakages in the deterministic system (with a mean 3.6 h^−1^). (*b*) Typical kinetics of the relative concentrations of *LR* and *PB* for the same simulation as (*a*). (*c*,*d*) Typical kinetics of *LR* and *PB* for noise with a mean 6.8 h^−1^ (*c*) and 7.2 h^−1^ (*d*). The inducer concentration (in relative units) is shown by magenta dotted lines.

Our simulations demonstrate that ITTS behaviour is very robust to variations in the P_ind_ strength (figure 4) and recombination efficiency (figure 6), while changes in P_1_ and P_2_ cause more drastic changes in the working range of the ITTS (figure 3). In particular, the leakages in P_1_ and P_2_ (i.e. expression from fully repressed promoters) strongly affect the bistability range of the ITTS (figure 3*c*). The levels of these leakages in P_1_ and P_2_ are expected to be noisy due to the low probability of RNA polymerase binding to P_1_ or P_2_ in the presence of high repressor concentrations. To simulate the potential effects of the noise on the ITTS kinetics, we replaced the leakages in P_1_ and P_2_ (parameter *k_tr_*_0_ in equations (2.1)–(2.4)) with the Poisson-distributed variables with a mean of 3.6 h^−1^ (equal to the leakages in the deterministic system) or 7.2 h^−1^ (in simulations with twofold increased noise). The noise was applied every minute. This results in noisy expression of *I*_1_, *I*_2_, *int* and *intRDF* proteins from P_1_ and P_2_. Our simulations demonstrate that even with relatively noisy leakages (with a mean of 3.6 h^−1^, figure 7*a*) the switch between the *PB* and *LR* states is robust to the noise (figure 7*b*). However, a further increase of the noise destabilizes the switching (figure 7*c*), leading to unpredictable switching when the noise is twofold higher than leakages in the deterministic system (figure 7*d*).

## 4. Conclusion

We present here a mathematical model of a single-input binary switch (ITTS), formed by combining a TTS and an inversion switch based on serine integrase-mediated site-specific recombination. The model predicts that the combined bistability of the TTS and unidirectionality of integrase-mediated recombination ensures nearly 100% efficiency of switching between two DNA states using repeated pulses of a single inducer. The ITTS is predicted to be robust to parameter perturbations and molecular noise. We envision that several ITTS modules built with orthogonal recombinases and repressors could be connected together sequentially to form a binary ‘ripple counter'. Each module represents a single binary digit and would signal the next module with a pulse of integrase expression every time it makes the transition from *LR* to *PB*. This would generate a counter, which would count sequentially through all binary numbers, to keep track of potentially large numbers of inter- or extracellular events [2].

## Supplementary Material

Supplementary figures, table and model code

## Data accessibility

There are no data associated with this paper.
