# Supplementary material for: Mathematical model of a serine integrase-controlled toggle switch with a single input
Source: J R Soc Interface. 2018 Jun 6;15(143):20180160. doi: 10.1098/rsif.2018.0160 (PMC6030632; doi:10.1098/rsif.2018.0160)
Supplement: Supplementary figures, table and model code [file rsif20180160supp1.doc]

**Supplementary Materials**

Alexandra Pokhilko, Oliver Ebenhöh, W. Marshall Stark and Sean D. Colloms

***Mathematical model of a serine integrase-controlled toggle switch with a single input***

Journal of the Royal Society Interface, 2018

*Supplementary Figure S1*

Schematic representations of *int*-mediated recombination reactions. *Int* converts *PB* substrate to *LR* product (red arrows), whereas *intRDF* converts *LR* to *PB* (blue arrows). *PBint, LRint_1_, LRint_2_, LRintRDF, PBintRDF_1_* and *PBintRDF_2_* are complexes containing 4 molecules of *int* or *intRDF* with *PB* or *LR*. The *PB*-to-*LR* conversion starts with the binding of 4 molecules of *int* to *PB*, followed by a recombination step and formation of *LRint_1_* product synapse. The favourable directions of reaction steps are shown by big arrowheads. The *LR*-to-*PB* conversion starts with the binding of 4 molecules of *intRDF* to *LR*, followed by a recombination step and formation of *PBintRDF_1_* product synapse. The *LRint_1_* and *PBintRDF_1_* products can very slowly change conformation (grey arrows), to give the *LRint_2_* and *PBintRDF_2_* complexes, which can release free *LR* and *PB* products. The main product complexes *LRint_1_* and *PBintRDF_1_* are shown in bold, and dotted lines show the main direction of the reactions. Step names are shown near arrows. The formation of unproductive complexes *LRintRDF_i_* and *PBintRDF_i_* , which each include one *int* dimer and one *intRDF* dimer, is not shown for clarity of the figure. The scheme is redrawn from .

*Supplementary Figure S2*

Scheme of a hypothetical one-input invertible switch. The switch expresses *intRDF* in the *PB* state and *int* in the *LR* state, thus maintaining the current state. A pulse of an inducer activates the inducible promoter P_ind_ and stimulates expression of the unexpressed recombinase (*int* or *intRDF*), which was expected to switch from the current to the alternate state

*Supplementary Figure S3*

| A | B |
| --- | --- |
| C  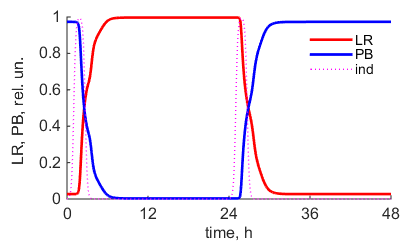 | D  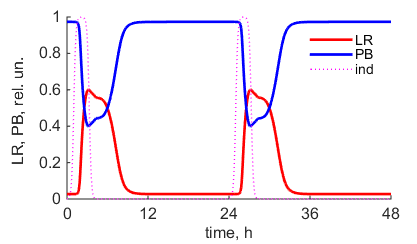 |

The kinetics of the switch with increased production of *int* and *intRDF* proteins, and pulse lengths inside (A,C) and outside (B,D) of the functional range. Simulations were done for a 30-fold increase of the rate of *int* and *intRDF* production and inducer pulse durations of 2.7 (A,C) and 3.3 (B,D) hours. A,B. The concentrations of *I_1_* (blue), *I_2_* (orange), *int* (green) and *intRDF* (black). The inserts show the kinetics during first 6 h. C,D. The relative concentrations of *LR* (red) and *PB* (blue). The inducer kinetics is shown on all panels by magenta dotted lines.

*Supplementary Table S1*

Table S1. Parameter values of the model.

| parameter | *k_+r_* | *k_-r1_* | *k_-r2_* | *k_+syn_* | *k_-syn_* |
| --- | --- | --- | --- | --- | --- |
| value | 6 h^-1^ | 2.14 h^-1^  (*K^eq^_r1_*=2.8) | 3 h^-1^  (*K^eq^_r2_*=2) | 0.006 h^-1^ | 0.017 h^-1^  (*K^eq^_syn_*=0.35) |
| ref. |  |  |  |  |  |
| parameter | *k_+synr_* | *k_-synr_* | *K^d^_bI_* | *K^d^_LRi_* | *k_t_* |
| value | 0.06 h^-1^ | 0.12 h^-1^  (*K^eq^_synr_* =0.5) | 0.0001 µM^4^ | 0.00002 µM^4^ | 0.3 h |
| ref |  |  |  |  | * |
| parameter | *k_tr_* | *k_tr0_* | *K_i_* | *k_dil_* |  |
| value | 360 h^-1^ | 3.6 h^-1^ | 0.01 µM | 2 h^-1^ |  |
| ref |  |  |  | * |  |

^*^ estimated based on 20 min doubling time

*Matlab code of the model*

**model file for deterministic simulations togg.m**

% main model file, calling for ode file Model_togg.m

Kr1=2.8; Kr2=2; Ksyn=0.36; Ksynr=0.5; KbI=0.0001; Klri=0.00002;

Dtot=0.01; k_dil=2; k_tr=360; k_tr0=3.6; Ki=0.01;

period=24; ind_on=1; ind_off=4;

% time unit - hour; concentrations - mkM

% y(1) BPtot

% y(2) LR-int4 first

% y(3) BP-int4-rdf4 first

% y(4) integrase

% y(5) RDF

% y(6) I1 prot

% y(7) I2 prot

y0=[0.00972964463211273,6.50549545377516e-08,0.000532655234484216,0.0190943801790866,0.405433920800401,0.405433920800401,0.0190943801790866;];

%options = odeset();

options = odeset('MaxStep',0.1);

t=[0 24*4]; % time interval

[T, Y] = ode15s(@Model_togg,t,y0,options,Dtot,Kr1,Kr2,Ksyn,Ksynr,KbI,Klri,k_tr,k_dil,Ki,k_tr0,ind_on,ind_off,period);

pulse=[ ];

kt=0.3;

for i=1:length(T)

Th(i)=T(i);

pulse(i)=0.5*(tanh((Th(i)-period*floor(Th(i)/period)-ind_on)/kt)-tanh((Th(i)-period*floor(Th(i)/period)-ind_off)/kt));

end

LRt=Dtot-Y(:,1);

figure (1)

plot(T,Y(:,6),'b');

hold on;

plot(T,Y(:,7),'y');

hold on;

plot(T,Y(:,4),'g');

hold on;

plot(T,Y(:,5),'k');

hold on;

plot(T,pulse,'m:');

hold on;

xlabel('time, h');

ylabel('I_1-green; I_2-red; int-blue; rdf-black, ind-mag');

figure (2)

plot(T,LRt/Dtot,'r');

hold on;

plot(T,Y(:,1)/Dtot,'b');

hold on;

plot(T,pulse,'m:');

hold on;

xlabel('time, h');

ylabel('LR_t-red; BP_t-blue; ind-mag');

figure (6)

plot(Y(:,6),Y(:,7),'k');

hold on;

title('phase diagram');

xlabel('I_1');

ylabel('I_2');

**the program uses the following function Model_togg.m:**

function Func = Model_togg(t,y,Dtot,Kr1,Kr2,Ksyn,Ksynr,KbI,Klri,k_tr,k_dil,Ki,k_tr0,ind_on,ind_off,period);

% solving ODEs

Func = zeros(7, 1);

kt=0.3;

puls=0.5*(tanh((t-period*floor(t/period)-ind_on)/kt)-tanh((t-period*floor(t/period)-ind_off)/kt));

% y(1) BPtot

% y(2) LR-int4 first

% y(3) BP-int4-rdf4 first

% y(4) integrase

% y(5) int-RDF

% y(6) I1 prot

% y(7) I2 prot

kpr=6;

kmr1=kpr/Kr1;

kmr2=kpr/Kr2;

kpsyn=0.006;

kmsyn=kpsyn/Ksyn;

kpsynr=0.06;

kmsynr=kpsynr/Ksynr;

Lr_t=Dtot-y(1);

Bp=(y(1)-y(3))/(1+y(4)^4/KbI+y(5)^4/KbI+y(4)^2*y(5)^2/KbI);

Lr=(Dtot-y(1)-y(2))/(1+y(4)^4/KbI+y(5)^4/KbI+y(4)^2*y(5)^2/Klri);

BpI=Bp*y(4)^4/KbI;

LrI2=Lr*y(4)^4/KbI;

LrIR=Lr*y(5)^4/KbI;

BpIR2=Bp*y(5)^4/KbI;

f1=1; % fold I2-P

f2=1; % fold leakagas

f3=1; % fold int, intRDF translation

f4=1; % fold Pind

f5=1; % fold I2-P leakage

f6=1; % fold I1-P leakage

f7=1; % fold I1-P

Func(1) = kmr1*y(2)-kpr*BpI+kpr*LrIR-kmr2*y(3);

Func(2) = kpr*BpI-kmr1*y(2)-kpsyn*y(2)+kmsyn*LrI2-k_dil*y(2);

Func(3) = kpr*LrIR-kmr2*y(3)-kpsynr*y(3)+kmsynr*BpIR2-k_dil*y(3);

Func(4) = k_tr*f3*f7*Dtot/(1+(y(6)/Ki)^2)+k_tr0*f2*f6*Dtot-k_dil*y(4);

Func(5) = k_tr*f3*f1*Dtot/(1+(y(7)/Ki)^2)+k_tr0*f2*f5*Dtot-k_dil*y(5);

Func(6) = k_tr*f4*puls*Lr_t+k_tr*f1*Dtot/(1+(y(7)/Ki)^2)+k_tr0*f2*f5*Dtot-k_dil*y(6);

Func(7) = k_tr*f4*puls*y(1)+k_tr*f7*Dtot/(1+(y(6)/Ki)^2)+k_tr0*f2*f6*Dtot-k_dil*y(7);

**model file for stochastic simulations togg_stoch_f.m**

% main model file, calling for ode file Model_togg_stoch.m

Kr1=2.8; Kr2=2; Ksyn=0.36; Ksynr=0.5; KbI=0.0001; Klri=0.00002;

Dtot=0.01; k_dil=2; k_tr=360; k_tr0=3.6; Ki=0.01;

period=24; ind_on=1; ind_off=4;

% time unit - hour; concentrations - mkM

% y(1) BPtot

% y(2) LR-int4 first

% y(3) BP-int4-rdf2 first

% y(4) integrase

% y(5) int-RDF

% y(6) I1 prot

% y(7) I2 prot

y0=[0.00972964463211273,6.50549545377516e-08,0.000532655234484216,0.0190943801790866,0.405433920800401,0.405433920800401,0.0190943801790866;];

%options = odeset();

options = odeset('MaxStep',0.1);

Tm=24*4*60; % max time in min

t0=0; T0=[]; T=[]; Y=[];

n=3.6; %n=6.8; % noise mean

for j=1:Tm

t=[(j-1)/60 j/60]; % time interval

R1 = poissrnd(n);

R2 = poissrnd(n);

R3 = poissrnd(n);

R4 = poissrnd(n);

[T1, Y1] = ode15s(@Model_togg_stoch_1217,t,y0,options,Dtot,Kr1,Kr2,Ksyn,Ksynr,KbI,Klri,k_tr,k_tr0,k_dil,Ki,ind_on,ind_off,period,R1,R2,R3,R4);

y0=[Y1(end,1) Y1(end,2) Y1(end,3) Y1(end,4) Y1(end,5) Y1(end,6) Y1(end,7)];

a=length(T0);

for i=1:length(T1)

T0(i+a)=T1(i);

for l=1:7

Y(i+a,l)=Y1(i,l);

end

end

end

T=T0';

pulse=[ ];

kt=0.3;

for i=1:length(T)

Th(i)=T(i);

pulse(i)=0.5*(tanh((Th(i)-period*floor(Th(i)/period)-ind_on)/kt)-tanh((Th(i)-period*floor(Th(i)/period)-ind_off)/kt));

end

LRt=Dtot-Y(:,1);

figure (1)

plot(T,Y(:,6),'b');

hold on;

plot(T,Y(:,7),'y');

hold on;

plot(T,Y(:,4),'g');

hold on;

plot(T,Y(:,5),'k');

hold on;

plot(T,pulse,'m:');

hold on;

xlabel('time, h');

ylabel('I_1-green; I_2-red; int-blue; rdf-black, ind-mag');

figure (2)

plot(T,LRt/Dtot,'r');

hold on;

plot(T,Y(:,1)/Dtot,'b');

hold on;

plot(T,pulse,'m:');

hold on;

xlabel('time, h');

ylabel('LR_t-red; BP_t-blue; ind-mag');

figure (3)

plot(tst/60,v_int,'b');

hold on;

plot(tst/60,v_intRDF,'k');

hold on;

plot(tst/60,v_I1,'g');

hold on;

plot(tst/60,v_I2,'r');

hold on;

plot(tst/60,Puls,'m:');

hold on;

**the program uses the following function Model_togg_stoch_1217.m:**

function Func = Model_togg_stoch_1217(t,y,Dtot,Kr1,Kr2,Ksyn,Ksynr,KbI,Klri,k_tr,k_tr0,k_dil,Ki,ind_on,ind_off,period,R1,R2,R3,R4);

% solving ODEs

Func = zeros(7, 1);

kt=0.3;

puls=0.5*(tanh((t-period*floor(t/period)-ind_on)/kt)-tanh((t-period*floor(t/period)-ind_off)/kt));

% y(1) BPtot

% y(2) LR-int4 first

% y(3) BP-int4-rdf2 first

% y(4) integrase

% y(5) int-RDF

% y(6) I1 prot

% y(7) I2 prot

kpr=6;

kmr1=kpr/Kr1;

kmr2=kpr/Kr2;

kpsyn=0.006;

kmsyn=kpsyn/Ksyn;

kpsynr=0.06;

kmsynr=kpsynr/Ksynr;

Lr_t=Dtot-y(1);

Bp=(y(1)-y(3))/(1+y(4)^4/KbI+y(5)^4/KbI+y(4)^2*y(5)^2/KbI);

Lr=(Dtot-y(1)-y(2))/(1+y(4)^4/KbI+y(5)^4/KbI+y(4)^2*y(5)^2/Klri);

BpI=Bp*y(4)^4/KbI;

LrI2=Lr*y(4)^4/KbI;

LrIR=Lr*y(5)^4/KbI;

BpIR2=Bp*y(5)^4/KbI;

Func(1) = kmr1*y(2)-kpr*BpI+kpr*LrIR-kmr2*y(3);

Func(2) = kpr*BpI-kmr1*y(2)-kpsyn*y(2)+kmsyn*LrI2-k_dil*y(2);

Func(3) = kpr*LrIR-kmr2*y(3)-kpsynr*y(3)+kmsynr*BpIR2-k_dil*y(3);

Func(4) = k_tr*Dtot/(1+(y(6)/Ki)^2)+R1*Dtot-k_dil*y(4);

Func(5) = k_tr*Dtot/(1+(y(7)/Ki)^2)+R2*Dtot-k_dil*y(5);

Func(6) = k_tr*puls*Lr_t+k_tr*Dtot/(1+(y(7)/Ki)^2)+R3*Dtot-k_dil*y(6);

Func(7) = k_tr*puls*y(1)+k_tr*Dtot/(1+(y(6)/Ki)^2)+R4*Dtot-k_dil*y(7);

*References*

[1] Pokhilko, A., Zhao, J., Stark, W.M., Colloms, S.D. & Ebenhöh, O. 2017 A simplified mathematical model of directional DNA site-specific recombination by serine integrases. *Journal of the Royal Society Interface* **14**. (doi:10.1098/rsif.2016.0618).

[2] Hooshangi, S., Thiberge, S. & Weiss, R. 2005 Ultrasensitivity and noise propagation in a synthetic transcriptional cascade. *Proc. Natl. Acad. Sci. U S A* **102**, 3581-3586. (doi:10.1073/pnas.0408507102).

[3] Elowitz, M.B. & Leibler, S. 2000 A synthetic oscillatory network of transcriptional regulators. *Nature* **403**, 335-338. (doi:10.1038/35002125).

[4] Biliouris, K., Daoutidis, P. & Kaznessis, Y.N. 2011 Stochastic simulations of the tetracycline operon. *BMC systems biology* **5**, 9. (doi:10.1186/1752-0509-5-9).
